# Linking Data From Health Surveys and Electronic Health Records: A Demonstration Project in Two Chicago Health Center Clinics

**DOI:** 10.5888/pcd15.170085

**Published:** 2018-01-18

**Authors:** Fikirte Wagaw, Catherine A. Okoro, Sunkyung Kim, Jessica Park, Fred Rachman

**Affiliations:** 1 Alliance of Chicago Community Health Services, Chicago, Illinois; 2 Division of Population Health, Centers for Disease Control and Prevention, Atlanta, Georgia; 3 Northrop Grumman, Atlanta, Georgia

## Abstract

**Introduction:**

Monitoring and understanding population health requires conducting health-related surveys and surveillance. The objective of our study was to assess whether data from self-administered surveys could be collected electronically from patients in urban, primary-care, safety-net clinics and subsequently linked and compared with the same patients’ electronic health records (EHRs).

**Methods:**

Data from self-administered surveys were collected electronically from a convenience sample of 527 patients at 2 Chicago health centers from September through November, 2014. Survey data were linked to EHRs.

**Results:**

A total of 251 (47.6%) patients who completed the survey consented to having their responses linked to their EHRs. Consenting participants were older, more likely to report fair or poor health, and took longer to complete the survey than those who did not consent. For 8 of 18 categorical variables, overall percentage of agreement between survey data and EHR data exceeded 80% (sex, race/ethnicity, pneumococcal vaccination, self-reported body mass index [BMI], diabetes, high blood pressure, medication for high blood pressure, and hyperlipidemia), and of these, the level of agreement was good or excellent (κ ≥0.64) except for pneumococcal vaccination (κ = 0.40) and hyperlipidemia (κ = 0.47). Of 7 continuous variables, agreement was substantial for age and weight (concordance coefficients ≥0.95); however, with the exception of calculated survey BMI and EHR–BMI (concordance coefficient = 0.88), all other continuous variables had poor agreement.

**Conclusions:**

Self-administered and web-based surveys can be completed in urban, primary-care, safety-net clinics and linked to EHRs. Linking survey and EHR data can enhance public health surveillance by validating self-reported data, completing gaps in patient data, and extending sample sizes obtained through current methods. This approach will require promoting and sustaining patient involvement.

## Introduction

Monitoring and understanding population health requires conducting health-related surveys and surveillance. The Behavioral Risk Factor Surveillance System (BRFSS), for example, is a state-based system of telephone surveys that collect data on health-risk behaviors, chronic conditions, use of preventive services, and health-related quality of life (HRQoL) of adults (1). BRFSS can be modified to assess emerging and urgent health issues and provides data on measures typically unrecorded in the clinical setting (eg, exercise, HRQoL, health attitudes, awareness, health knowledge) (2,3). Searching for new data sources is important, however, because population-based surveys can be costly and time-consuming and may produce biased results that are hard to generalize (1,4–12).

Expanded use of electronic health records (EHRs) — complete with appropriate protection of patient confidentiality — can help improve the design and delivery of public health interventions and clinical care; data in EHRs can be used to help find new causes of infectious disease and to address outbreaks by triggering public health alerts, providing recommendations to clinicians, and enhancing communications between public health practitioners and clinical organizations (11–13). Additionally, EHRs can help identify patients needing medical care, disease management, preventive health services, and behavioral counseling (2,3,14–17). EHRs can also help control rising health care costs by eliminating unnecessary tests, procedures, and prescriptions (17).

EHRs may help improve patient care and population health when linked to survey data and other information about health-related behavior, HRQoL, and details about working and living conditions (2,3,18). For people managing a chronic illness, for example, the EHR can validate responses, because survey answers can be linked to recorded clinical events. Likewise, behaviors (eg, exercise) recorded in a recent survey could trigger alerts and recommendations back through the EHR. Inclusion of patient-reported measures in EHRs can enhance patient-centered care, patient health, and capacity to conduct population-based research (2,3).

The objective of this study was to explore the feasibility of electronically collecting self-administered patient survey data in urban, primary-care, safety-net clinics and subsequently linking and comparing that data with patients' EHR data.

## Methods

Alliance of Chicago Community Health Services (Alliance; http://alliancechicago.org/) is a federal Health Center Controlled Network. Alliance approached 4 of its network health centers about project participation, selecting them for their large patient volume, diverse geographic locations, distinct and diverse patient populations, and history of participation in new initiatives. Although 3 health centers approved the project, only 1 was able to participate in the project’s timeframe. We implemented our study in 2 of that health center’s clinics, and it was approved by that clinic’s research review committee.

We recruited clinic patients aged 18 years or older by using fliers and announcements in waiting areas and in check-in procedures. Survey administrators used standardized scripts to summarize the survey’s goals for interested patients. Participants reviewed an electronic consent form and received a hard copy of the form; they provided separate informed consent for survey participation and for subsequent survey–EHR linkage. Each survey participant received a modest incentive (regardless of consent to EHR linkage). Survey administrators were available to assist patients throughout data collection.

From September 2014 through November 2014, a convenience sample of 527 patients completed the self-administered, web-based survey on various brands of electronic tablets, desktop computers, and cellular phones. Tablet data plans were purchased to minimize impact on health center resources and to minimize data connectivity issues.

Questions from the Illinois BRFSS (http://app.idph.state.il.us/brfss/) were used to collect information on patients’ sociodemographic characteristics, health behaviors, chronic conditions, receipt of preventive care services, and medical care. Questions related to chronic conditions were selected on the basis of their ability to be matched to data available in the EHR. Questions on medication use, laboratory findings, and blood pressure readings helped us compare data on self-reported chronic conditions with EHR content. The number of questions each participant was asked was determined by sex (eg, sex-specific preventive care services), age (eg, age-specific cancer screenings), and survey responses that determined question branching. The survey took an average of 20 to 30 minutes to complete and was hosted by using the Survey Analytics Online Survey Platform (Survey Analytics LLC).

Of 527 survey participants, 47% (n = 251) consented to have their survey responses linked to their EHR; 99% (n = 248) of these consenting patients had an EHR. At the end of the survey and EHR extraction, 2 de-identified analytic data sets were created: 1) a set that contained only the survey data of patients who did not consent to the EHR linkage and 2) a set that contained the survey and EHR data of patients who consented to EHR linkage.

When possible, differences in categorical variable construction between survey data and EHR data were resolved by collapsing the original categories to form a common metric. Continuous variables except for blood pressure were constructed similarly in the survey instrument and the EHR. Patients reporting that a health care professional said they had high blood pressure (HBP) were asked to enter their systolic and diastolic blood pressure. For the EHR abstraction, the last 3 systolic and diastolic blood pressure readings were taken, and the mean systolic and diastolic pressures were calculated. Self-reported weight and height were assessed using 2 survey questions: “About how much do you weigh without shoes?” and “About how tall are you without shoes?” Patients were classified as underweight (body mass index [BMI, kg/m^2^] <18.5), normal weight (BMI 18.5–<25), overweight (BMI 25–<30), or obese (BMI ≥30). Self-classified BMI was assessed with the survey question, “Would you classify your weight as low (underweight), normal weight, overweight, or obese?”

We calculated the distribution of the study population by survey duration, sociodemographic characteristics, and self-rated health status, overall and by consent to EHR linkage. For categorical variables, we used the χ^2^ test to assess significant differences between those who agreed to survey–EHR linkage and those who did not. For continuous variables, we used the *t* test to assess significant mean differences between the 2 patient groups. To assess concordance between survey data and EHR data, we examined 248 patients who consented to the EHR linkage and for whom an EHR record was found. For categorical variables, we applied Cohen’s (19) κ coefficient with 4 predefined agreement levels: excellent agreement (κ ≥0.9), good agreement (κ ≥0.6 to κ <0.9), fair agreement (κ ≥0.3 to κ <0.6), and poor agreement (κ <0.3). Because we observed some cases that may belong to the κ paradox (20), we also calculated overall agreement in percentage (= 100 × the number of concordant counts/the total sample size). For continuous variables, we applied Lin’s (21,22) concordance correlation coefficient (*ρ_c_*) with 4 predefined agreement levels: almost perfect (*ρ_c_* >0.99), substantial (*ρ_c_* ≥0.95 to *ρ_c_* ≤0.99), moderate (*ρ_c_* ≥0.90 to *ρ_c_* <0.95), and poor (*ρ_c_* <0.90) (23). For all analyses, *P* < .05 was considered significant, and data were analyzed in SAS version 9.3 (SAS Institute, Inc).

## Results

Participant ages ranged from 18 to 87 years (mean, 43.4 y; standard deviation [SD], 14.7 y) (Table 1). The sample was predominantly non-Hispanic black (90.4%), female (70.4%), never married or a member of an unmarried couple (61.2%), spoke English as their primary language (96.3%), and had Medicaid or Medicare as primary health insurance coverage (69.1%). More than 70% reported their health status as excellent, very good, or good, and 62.7% reported no disability. Most had annual household incomes less than $20,000, rented their primary residence, and had no children in the household.

**Table 1:** Demographic Characteristics of Study Population^a^, by Agreement to Survey and EHR Data Linkage, 2 Chicago Health Centers, 2014

Characteristic	Overall	EHR Linkage		*P* Value^c^
		Yes	No	
**Total^b^**	527 (100)	251 (47.6)	276 (52.4)	
**Survey duration, no. min, (mean) [SD]**	509 (24.4) [17.0]	245 (27.8) [18.6]	264 (21.3) [14.8]	<.001
**Age, y, no. (mean) [SD]**	507 (43.4) [14.7]	244 (45.4) [14.6]	263 (41.5) [14.6]	.003
**Age group, y**				
18–34	168 (33.1)	72 (29.5)	96 (36.5)	.01
35–44	83 (16.4)	31 (12.7)	52 (19.8)	
45–64	227 (44.8)	125 (51.2)	102 (38.8)	
≥65	29 (5.7)	16 (6.6)	13 (4.9)	
**Sex**				
Male	149 (29.6)	64 (26.2)	85 (32.8)	.11
Female	354 (70.4)	180 (73.8)	174 (67.2)	
**Race/ethnicity**				
Non-Hispanic black	424 (90.4)	202 (89.4)	222 (91.4)	.91
Hispanic	26 (5.5)	14 (6.2)	12 (4.9)	
Non-Hispanic other^d^	13 (2.8)	7 (3.1)	6 (2.5)	
Non-Hispanic white	6 (1.3)	3 (1.3)	3 (1.2)	
**Education**				
<High school diploma	108 (21.6)	62 (25.5)	46 (17.8)	.05
High school diploma or GED	144 (28.7)	60 (24.7)	84 (32.6)	
>High school	249 (49.7)	121 (49.8)	128 (49.6)	
**Marital status**				
Married	72 (14.7)	35 (15.0)	37 (14.5)	.09
Divorced	53 (10.8)	30 (12.8)	23 (9.0)	
Separated	44 (9.0)	15 (6.4)	29 (11.4)	
Widowed	21 (4.3)	14 (6.0)	7 (2.8)	
Never married/member of an unmarried couple	299 (61.2)	140 (59.8)	159 (62.4)	
**Employment status**				
Employed	214 (42.5)	103 (42.2)	111 (42.7)	.85
Retired	32 (6.4)	19 (7.8)	13 (5.0)	
Student	43 (8.5)	19 (7.8)	24 (9.2)	
Homemaker	28 (5.6)	14 (5.7)	14 (5.4)	
Unable to work	60 (11.9)	28 (11.5)	32 (12.3)	
Unemployed	127 (25.2)	61 (25.0)	66 (25.4)	
**Disability** e				
Yes	150 (37.3)	84 (39.8)	66 (34.6)	.28
No	252 (62.7)	127 (60.2)	125 (65.5)	
**Primary language**				
English	472 (96.3)	232 (96.3)	240 (96.4)	.26
Spanish	9 (1.8)	6 (2.5)	3 (1.2)	
Spanish and English equally	8 (1.6)	2 (0.8)	6 (2.4)	
Other	1 (0.2)	1 (0.4)	0	
**Type of health coverage**				
Employer	33 (7.1)	14 (6.0)	19 (8.3)	.33
Medicaid/Medicare	320 (69.1)	166 (79.9)	154 (67.3)	.39
School	2 (0.4)	0	2 (0.9)	.15
Self-purchase	12 (2.6)	5 (2.1)	7 (3.1)	.53
None	98 (21.2)	50 (21.4)	48 (21.0)	.91
**General health**				
Fair/poor	135 (27.8)	76 (32.2)	59 (23.6)	.03
Excellent/very good/good	351 (72.2)	160 (67.8)	191 (76.4)	
**Household characteristics**				
**Annual household income, $**				
<10,000	191 (43.2)	88 (42.3)	103 (44.0)	.98
10,000–19, 999	114 (25.8)	55 (26.4)	59 (25.2)	
20,000–39,999	106 (24.0)	50 (24.0)	56 (23.9)	
≥40,000	31 (7.0)	15 (7.2)	16 (6.8)	
**Children younger than 18 years in household**				
None	269 (54.8)	134 (56.5)	135 (53.2)	.45
≥1	222 (45.2)	103 (43.5)	119 (46.9)	
**Household total members, no. (mean) [SD]**	488 (2.7) [1.8]	236 (2.6) [1.7]	252 (2.8) [2.0]	.24
Adults, mean (SD)	1.8 (1.0)	1.7 (0.9)	1.8 (1.1)	.35
Children younger than 18 years, mean (SD)	0.9 (1.4)	0.9 (1.3)	1.0 (1.5)	.38
**Home ownership**				
Own	53 (10.8)	25 (10.5)	28 (11.0)	.58
Rent	359 (73.0)	170 (71.4)	189 (74.4)	
Other arrangement^f^	80 (16.3)	43 (18.1)	37 (14.6)	

Abbreviation: EHR, electronic health record; GED, General Educational Development certificate; SD, standard deviation.

a Values are no. (%) unless otherwise indicated.

b Number of participants for whom data were available. Section numbers may not total 527 because of missing values.

c χ^2^ Test was used for categorical variables and *t* test was used for continuous variables to determine *P* values to test the difference between adults who agreed to the EHR linkage and those who did not.

d Includes respondents who reported their ethnicity as non-Hispanic and their race as American Indian or Alaska Native, Asian or Asian American, Native Hawaiian or Pacific Islander, mixed race, or some other race.

e Patients were categorized as having a disability if they responded yes to any of 6 questions on hearing disability, vision disability, cognitive disability, ambulatory disability, self-care disability, or independent living disability. Patients who answered no to all 6 questions were categorized as not having a disability.

f Other housing, such as group home or staying with friends or family without paying rent.

Seven health behaviors of the convenience sample were distributed as follows: always or nearly always wearing a seat belt (90.0%), watching or reducing sodium intake (60.2%), consuming one or more drinks of alcohol in the past 30 days (59.6%), engaging in leisure-time physical activity (58.3%), consuming 5 or more servings daily of fruits and vegetables (42.2%), currently smoking cigarettes (35.4%), and increasing medication use in the past 30 days without the advice of a health care professional (8.9%) (Figure).

**Figure:**
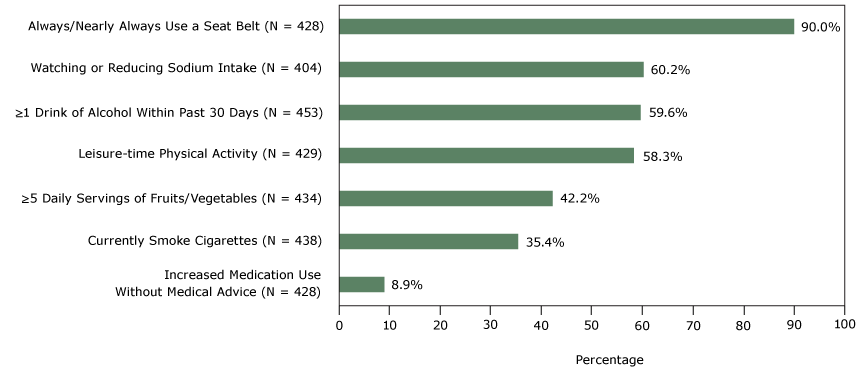
Self-reported health behaviors of a convenience sample, study linking self-reported survey data with electronic health record data, 2 Chicago health clinics, 2014. **Table:** 

Self-Reported Health Behavior	N (%)
Always or nearly always use a seat belt	428 (90.0)
Watch or reduce sodium intake	404 (60.2)
Consumed one or more alcoholic beverages within past 30 days	453 (59.6)
Engage in leisure-time physical activity	429 (58.3)
Consume 5 or more servings daily of fruits and vegetables	434 (42.2)
Currently smoke cigarettes	438 (35.4)
Increased medication use without medical advice within past 30 days	428 (8.9)

Compared with patients who did not agree to having their survey results linked to their EHRs, those who agreed were older (mean 45.4 y vs 41.5 y, *P* = .003), more likely to report fair or poor health (32% vs 24%, *P* = .03), and took longer to complete the electronic survey (27.8 minutes vs 21.3 minutes, *P* < .01) (Table 1).

Of the 18 categorical variables we examined, overall agreement between the survey and EHR data exceeded 80% for 8 variables (sex, race/ethnicity, pneumococcal vaccination, self-reported BMI, diabetes, HBP, medication for HBP, hyperlipidemia), and of these, the level of agreement was good or excellent (κ ≥ 0.64) except for pneumococcal vaccination (κ = 0.40) and hyperlipidemia (κ = 0.47) (Table 2). Race/ethnicity and diabetes had a percentage agreement above 91% but lower κ statistics values of 0.65 (95% confidence interval [CI], 0.45–0.85) and 0.76 (95% CI, 0.66–0.87), respectively. Self-classified BMI showed the lowest level of concordance (overall agreement = 19.5%, κ = 0.16).

**Table 2:** Measures of Concordance Between Data From Health Survey and EHR for Categorical Items Contained in Both Data Sources, 2 Chicago Health Center Clinics, 2014

Variable	Data Source		Measures of Concordance	
	Survey N (%)	EHR N (%)	Overall Agreement, %^a^	κ (95% CI)^a^
**Sex (n = 241)**				
Male	62 (25.7)	63 (26.1)	99.6	0.99 (0.97 to 1.00)
Female	179 (74.3)	178 (73.9)		
**Race/ethnicity (n = 217)**				
Non-Hispanic black	197 (90.8)	207 (95.4)	95.4	0.65 (0.45 to 0.85)
Hispanic	13 (6.0)	9 (4.2)		
Non-Hispanic other^b^	7 (3.2)	1 (0.5)		
Non-Hispanic white	0	0		
**Education (n = 40)**				
<High school diploma	7 (17.5)	5 (12.5)	65.0	0.44 (0.23 to 0.64)
High school/GED	8 (20.0)	18 (45.0)		
>High school diploma	25 (62.5)	17 (42.5)		
**Marital status (n = 229)**				
Married	33 (14.4)	27 (11.8)	67.5	0.33 (0.23 to 0.43)
Divorced	29 (12.7)	4 (1.8)		
Separated	15 (6.6)	5 (2.2)		
Widowed	14 (6.1)	1 (0.4)		
Never married/member of an unmarried couple	138 (60.3)	192 (83.8)		
**Employment status (n = 211)**				
Employed	89 (42.2)	62 (29.4)	62.9	0.38 (0.28 to 0.48)
Retired	17 (8.1)	6 (2.8)		
Student	15 (7.1)	5 (2.4)		
Other^c^	90 (42.7)	138 (65.4)		
**Primary insurance (n = 171)** d				
Public	126 (73.7)	103 (60.2)	62.1	0.29 (0.16 to 0.42)
Employer	11 (6.4)	2 (1.2)		
None	34 (19.9)	66 (38.6)		
**Annual household income (n = 108), $**				
<10,000	34 (31.5)	48 (44.4)	37.0	0.08 (−0.05 to 0.20)
10,000–19,999	36 (33.3)	49 (45.4)		
20,000–39,999	32 (29.6)	10 (9.3)		
≥40,000	6 (5.6)	1 (0.9)		
**HPV DNA test (n = 109 women)**				
Yes	43 (39.5)	10 (9.2)	62.4	0.09 (−0.05 to 0.23)
No	66 (60.6)	99 (90.8)		
**Influenza shot/spray (n = 199)**				
Yes	76 (38.2)	46 (23.1)	71.9	0.36 (0.23 to 0.49)
No	123 (61.8)	153 (76.9)		
**Tetanus vaccination since 2005 (n = 198)**				
Yes	95 (48.0)	13 (6.6)	54.6	0.06 (−0.01 to 0.13)
No	103 (52.0)	185 (93.4)		
**Pneumococcal vaccine (n = 192)**				
Yes	42 (21.9)	26 (13.5)	82.3	0.40 (0.24 to 0.56)
No	150 (78.1)	166 (86.5)		
**Self-reported BMI, kg/m^2^ (n = 205)** e				
Underweight (<18.5)	5 (2.4)	5 (2.4)	86.3	0.78 (0.70 to 0.85)
Normal weight (18.5 – <25.0)	43 (21.0)	43 (21.0)		
Overweight (≤25.0 – <30.0)	48 (23.4)	41 (20.0)		
Obese (≥30.0)	109 (53.2)	116 (56.6)		
**Self-classified BMI (n = 210)** f				
Low (underweight)	18 (8.6)	5 (2.4)	19.5	0.16 (0.08 to 0.23)
Normal weight	75 (35.7)	46 (21.9)		
Overweight	95 (45.2)	41 (19.5)		
Obese	22 (10.5)	118 (56.2)		
**Diabetes (n = 212)**				
Yes	52 (24.5)	46 (21.7)	91.5	0.76 (0.66 to 0.87)
No	160 (75.5)	166 (78.3)		
**High blood pressure (n = 208)**				
Yes	109 (52.4)	87 (41.8)	84.6	0.69 (0.60 to 0.79)
No	99 (47.6)	121 (58.2)		
**Taking medication for high blood pressure (n = 108)**				
Yes	90 (83.3)	81 (75.0)	87.2	0.64 (0.46 to 0.81)
No	18 (16.7)	27 (25.0)		
**Hyperlipidemia (n = 201)**				
Yes	46 (22.9)	31 (15.4)	83.6	0.47 (0.32 to 0.63)
No	155 (77.1)	170 (84.6)		
**Taking medication for hyperlipidemia (n = 42)**				
Yes	25 (59.5)	30 (71.4)	75.0	0.53 (0.27 to 0.79)
No	17 (40.5)	12 (28.6)		

Abbreviations: BMI, body mass index; CI, confidence interval; EHR, electronic health record; GED, general equivalency degree; HBP, high blood pressure; HPV, human papillomavirus; N, number of eligible patients included in item-level analysis.

a Defined as the number of concordant counts (both answered yes or both answered no in 2 sources) divided by the total sample size and expressed as a percentage. κ ≥0.9 = excellent agreement, κ ≥0.6 to κ <0.9 = good agreement, κ ≥0.3 to κ <0.6 = fair agreement, and κ <0.3 = poor agreement.

b Includes respondents who reported their ethnicity as non-Hispanic and their race as American Indian or Alaska Native, Asian or Asian American, Native Hawaiian or Pacific Islander, mixed race, or some other race. In EHR data, patients coded as Hispanic or Latino did not have a race code. Similarly, patients with a race value did not have an ethnicity code.

c Includes unemployed, homemaker, and unable to work. Patients coded as unemployed in EHR are categorized as other.

d Patients who responded “Yes, through my school” or “Yes, I purchased on my own” on survey were not included in this analysis because EHR data did not have equivalent categories.

e Self-reported BMI was based on 2 survey questions: “About how much do you weigh without shoes?” and “About how tall are you without shoes?” and compared with the EHR’s BMI based on the EHR’s height and weight variables. Patients who responded “Don’t know/not sure” to either question or who were missing an EHR value were not included in this analysis.

f Self-classified BMI was based on the survey question, “Would you classify your weight as: low (underweight), normal, overweight, or obese?” and compared with calculated BMI based on the EHR’s height and weight variables. Patients who responded “Don’t know/not sure” or who were missing an EHR value were not included in this analysis.

Of the 7 continuous variables we examined, the agreement level between the survey and EHR data was substantial for age (= 0.95; 95% CI, 0.94–0.96) and weight (= 0.98; 95% CI, 0.97–0.98) (Table 3). With the exception of BMI (= 0.88; 95% CI, 0.84–0.91), all other continuous variables had poor agreement; diastolic blood pressure among patients who reported hypertension had the lowest agreement (= 0.28; 95% CI, 0.13–0.41).

**Table 3:** Measures of Concordance Between Survey and EHR for Continuous Items Contained in Both Data Sources, 2 Chicago Health Center Clinics, 2014

Item	No.^a^	Data Source		Concordance Correlation Coefficient, (95% CI)^b^
		Survey Mean (SD)	EHR Mean (SD)	
Age, y	241	45.5 (14.7)	45.6 (14.7)	0.95 (0.94–0.96)
Height, in	216	65.6 (4.8)	65.8 (4.1)	0.78 (0.73–0.83)
Weight, lbs	211	195.4 (54.3)	197.9 (55.6)	0.98 (0.97–0.98)
BMI, kg/m^2c^	205	31.8 (8.5)	32.0 (8.3)	0.88 (0.84–0.91)
Hemoglobin A1c^d^	11	7.5 (2.3)	8.2 (3.2)	0.68 (0.17–0.90)
Systolic BP, mm Hg^e^	55	133.0 (21.7)	133.6 (19.7)	0.60 (0.39–0.74)
Diastolic BP, mm Hg^e^	54	80.6 (13.6)	99.0 (14.7)	0.28 (0.13–0.41)

Abbreviation: BMI, body mass index; BP, blood pressure; CI, confidence interval; EHR, Electronic health record; N,; SD, standard deviation.

a Number of eligible patients included in item-level analysis.

b Substantial agreement = *ρ_c_* ≥0.95 to *ρ_c_* ≤0.99; poor agreement = *ρ_c_* <0.90.

c Self-reported BMI was based on 2 survey questions (“About how much do you weigh without shoes?” and “About how tall are you without shoes?”) and compared with EHR’s BMI based on EHR’s height and weight variables. Patients who responded “Don’t know/not sure” to either question or who were missing an EHR value were not included in this analysis.

d Last hemoglobin A1c among patients who reported being told by a health professional that they had diabetes.

e Among patients who reported being told by a health professional that they had high blood pressure.

## Discussion

This study compared results from a self-administered web-based survey with de-identified patient data from EHRs in an urban primary-care setting. We found a satisfactory degree of concordance between survey data and EHR data for nonmodifiable demographic characteristics and for some health-related measures: diabetes, HBP, HBP medication, weight, and calculated categorical and continuous BMI. We found lower levels of concordance for modifiable sociodemographic characteristics, pneumococcal vaccination, hyperlipidemia, self-classified BMI, hemoglobin A1c among patients reporting diabetes, and blood pressure among patients reporting hypertension ― especially diastolic pressure. EHR data on self-reported health-risk behaviors were unavailable for comparison; data on tobacco use screening were available.

Fewer than half the surveyed patients gave EHR linkage consent; those consenting showed significant differences from those who did not. Similar to other researchers’ findings (24), those consenting were older and more likely to report fair or poor health. Unlike other research findings (24), however, we did not find significant differences by sex, employment status, or type of health insurance coverage. Further investigation into what factors may increase consent or enhance patient engagement could aid project sustainability and representativeness of the patient population.

Generally, our concordance findings were consistent with studies that have used similar methods (7,25,26). Our level of agreement was similar to previous research assessing data quality between ambulatory medical record data and patient survey data for diabetes and BMI, but we had a higher level of concordance for HBP, HBP medication, and hyperlipidemia and a lower level of concordance for lipid-lowering medication (26). Additionally, we had substantial agreement for weight and, in contrast, poor agreement for height. We also found good overall agreement for BMI based on self-reported height and weight (86%) but poor overall agreement for self-classified BMI (20%). Studies show people generally overestimate their height and underestimate their weight and BMI (6). This reporting bias varies, however, by the demographic characteristics of the study population (eg, sex, race, age). For example, men are more likely to exaggerate their height than women are. Our convenience sample was predominantly female, non-Hispanic black, and aged 45 to 64 years. Differences between self-report and direct measures may also be due to the respective population’s sociocultural perceptions of body weight and may be biased by social desirability (6). Our results demonstrate the need for direct measures that validate self-reported data, because patients were more likely to perceive themselves as in a lower BMI category than their calculated BMI category showed. Our results may also reflect a lack of awareness of their BMI and, consequently, greater risk of poor health outcomes. Further research is needed to fully understand and address this finding (eg, improved patient–provider communication, obesity screening and intervention). Because our results are not generalizable to the health center’s patient population or to other patient populations (convenience sample/apparent selection bias), interpretation should be done with care.

Survey and EHR data may have poor concordance for many reasons and may show where each data source can help improve the accuracy and completeness of patient and population data. When survey and EHR clinical measures are not concordant, EHR data tend to be more accurate than survey data because biases associated with self-report vary (5–7). For example, correctly remembering the date of one’s last tetanus shot or hemoglobin A1c test result is difficult. For modifiable sociodemographic characteristics, however, self-reported data are likely to be more accurate than EHR data, because busy health centers have few resources or incentives to update nonclinical data elements. Institutional incentives also may influence poor concordance, as when a sliding fee scale could encourage under-reporting of income or private health care coverage or when health insurance plans charge higher premiums to consumers who smoke (27).

Our study has several limitations. First, we used a convenience sample of patients from 2 Chicago health center clinics. This sample selection bias limits our ability to make inferences to the health center’s patient population across all its clinic sites and its comparability to other patient populations in the area; the sample was predominantly female, non-Hispanic black, unmarried, and low income; patients had public insurance coverage and were more likely to have a cellular telephone or an email address than a landline telephone. As a result, for public health surveillance, multiple data collection modes and data sources may be needed to effectively reach and ensure the representativeness of data for population subgroups. Moreover, public health professionals and policy makers must be aware of subpopulations that are unconnected to the health care system and whose members have limited health records or lack them entirely (4). Second, less than 50% of the patients surveyed consented to EHR linkages. Further analysis of the factors associated with consent, and which are amenable to modification, is needed to access the wealth of data available in EHRs. Third, analysis of the linked data found some variables with low prevalence that prevented further assessment of agreement. Fourth, some variables had good agreement but low κ scores, suggesting that agreement may have occurred by chance alone. Finally, neither data source may be considered a gold standard for all items measured. For example, survey data may have inherent biases, and EHR data and the data extraction process may have complexities that are not fully known or accounted for. Nevertheless, these limitations may change over time with meaningful use of EHRs, advancements in health information technologies, and emphasis on quality and patient-centered care as well as implementing new methods that integrate lifestyle measures into prescribed health care (eg, prescribed physical activity) (2–4,28).

These limitations notwithstanding, a symbiotic relationship exists between survey data and clinical data. Self-reported data are needed to augment clinical data for medical services (eg, immunizations, screenings, behavioral counseling), imaging and other diagnostics, and medications obtained outside of the patient’s health center (2,7). Self-reported measures, although subject to biases, are vital to providing a complete picture of patient health, because many health-related measures may not be in the EHR (eg, behaviors, HRQoL, health attitudes, awareness, knowledge) or up-to-date (eg, modifiable sociodemographic characteristics) (2,17,29,30). At the same time, EHRs can be used to validate self-reported clinical measures and facilitate the development of correction factors that can be applied to self-reported data in the absence of physical measurement, which is often costly or not possible (6). In unison, the 2 data sources have the potential to improve disease management, reduce costs, and enhance two-way data exchange between public health and clinical organizations.

As health systems and their information technologies continue to evolve, researchers should continue the search for high-quality patient health data. Doing so can help health practitioners, public health professionals, and policy makers successfully evaluate and reduce existing health disparities. Furthermore, public health policy and practice can be guided by data science methods (including predictive analytics) by using combined data sources. Population-based surveys, EHRs, and other data sources all have a role in providing a more complete picture of the health of all Americans, while improving their health and access to care. To this end, this project demonstrated the feasibility of computer-assisted collection of consumer survey data and matching it to EHR data. This approach can enhance health information from unique, often underrepresented populations with health disparities, increase efficiency and breadth of surveillance activities, and improve validity of objective measures. More research is needed to promote and sustain patient involvement in their health and health records, which is vital to the success and sustainability of this approach.

## References

[1] Pierannunzi C , Hu SS , Balluz L . A systematic review of publications assessing reliability and validity of the Behavioral Risk Factor Surveillance System (BRFSS), 2004–2011. BMC Med Res Methodol 2013;13(1):49. 10.1186/1471-2288-13-49 23522349 PMC3622569

[2] Glasgow RE , Kaplan RM , Ockene JK , Fisher EB , Emmons KM . Patient-reported measures of psychosocial issues and health behavior should be added to electronic health records. Health Aff (Millwood) 2012;31(3):497–504. 10.1377/hlthaff.2010.1295 22392660

[3] Krist AH , Phillips SM , Sabo RT , Balasubramanian BA , Heurtin-Roberts S , Ory MG , ; MOHR Study Group. Adoption, reach, implementation, and maintenance of a behavioral and mental health assessment in primary care. Ann Fam Med 2014;12(6):525–33. 10.1370/afm.1710 25384814 PMC4226773

[4] Crilly JF , Keefe RH , Volpe F . Use of electronic technologies to promote community and personal health for individuals unconnected to health care systems. Am J Public Health 2011;101(7):1163–7. 10.2105/AJPH.2010.300003 21566023 PMC3110234

[5] Cronin KA , Miglioretti DL , Krapcho M , Yu B , Geller BM , Carney PA , Bias associated with self-report of prior screening mammography. Cancer Epidemiol Biomarkers Prev 2009;18(6):1699–705. 10.1158/1055-9965.EPI-09-0020 19505902 PMC2771779

[6] Connor Gorber S , Tremblay M , Moher D , Gorber B . A comparison of direct vs. self-report measures for assessing height, weight and body mass index: a systematic review. Obes Rev 2007;8(4):307–26. 10.1111/j.1467-789X.2007.00347.x 17578381

[7] Rolnick SJ , Parker ED , Nordin JD , Hedblom BD , Wei F , Kerby T , Self-report compared to electronic medical record across eight adult vaccines: do results vary by demographic factors? Vaccine 2013;31(37):3928–35. 10.1016/j.vaccine.2013.06.041 23806243 PMC4689428

[8] Khoury MJ , Lam TK , Ioannidis JP , Hartge P , Spitz MR , Buring JE , Transforming epidemiology for 21st century medicine and public health. Cancer Epidemiol Biomarkers Prev 2013;22(4):508–16. 10.1158/1055-9965.EPI-13-0146 23462917 PMC3625652

[9] Gittelman S , Lange V , Gotway Crawford CA , Okoro CA , Lieb E , Dhingra SS , A new source of data for public health surveillance: Facebook likes. J Med Internet Res 2015;17(4):e98. 10.2196/jmir.3970 25895907 PMC4419195

[10] Vogel J , Brown JS , Land T , Platt R , Klompas M . MDPHnet: secure, distributed sharing of electronic health record data for public health surveillance, evaluation, and planning. Am J Public Health 2014;104(12):2265–70. 10.2105/AJPH.2014.302103 25322301 PMC4232140

[11] Smolinski MS , Crawley AW , Baltrusaitis K , Chunara R , Olsen JM , Wójcik O , Flu Near You: crowdsourced symptom reporting spanning 2 influenza seasons. Am J Public Health 2015;105(10):2124–30. 10.2105/AJPH.2015.302696 26270299 PMC4566540

[12] Smart Chicago Collaborative. Foodborne Chicago; 2017. http://www.smartchicagocollaborative.org/people/staff-consultants/. Accessed November 20, 2017.

[13] Lurio J , Morrison FP , Pichardo M , Berg R , Buck MD , Wu W , Using electronic health record alerts to provide public health situational awareness to clinicians. J Am Med Inform Assoc 2010;17(2):217–9. 10.1136/jamia.2009.000539 20190067 PMC3000778

[14] Moody-Thomas S , Nasuti L , Yi Y , Celestin MD Jr , Horswell R , Land TG . Effect of systems change and use of electronic health records on quit rates among tobacco users in a public hospital system. Am J Public Health 2015;105(S2, Suppl 2):e1–7. 10.2105/AJPH.2014.302274 25689197 PMC4355704

[15] Kern LM , Barrón Y , Dhopeshwarkar RV , Edwards A , Kaushal R ; HITEC Investigators. Electronic health records and ambulatory quality of care. J Gen Intern Med 2013;28(4):496–503. 10.1007/s11606-012-2237-8 23054927 PMC3599037

[16] Murphy DR , Laxmisan A , Reis BA , Thomas EJ , Esquivel A , Forjuoh SN , Electronic health record-based triggers to detect potential delays in cancer diagnosis. BMJ Qual Saf 2014;23(1):8–16. 10.1136/bmjqs-2013-001874 23873756

[17] Office of the National Coordinator for Health Information Technology (ONC), Department of Health and Human Services. 2014 Edition Release 2 Electronic Health Record (EHR) certification criteria and the ONC HIT Certification Program; regulatory flexibilities, improvements, and enhanced health information exchange. Final rule. Fed Regist 2014;79(176):54429–80. 25233533

[18] Gustafsson PE , San Sebastian M , Janlert U , Theorell T , Westerlund H , Hammarström A . Life-course accumulation of neighborhood disadvantage and allostatic load: empirical integration of three social determinants of health frameworks. Am J Public Health 2014;104(5):904–10. 10.2105/AJPH.2013.301707 24625161 PMC3987591

[19] Cohen J . A coefficient of agreement for nominal scales. Educ Psychol Meas 1960;20(1):37–46. 10.1177/001316446002000104

[20] Cicchetti DV , Feinstein AR . High agreement but low kappa: II. Resolving the paradoxes. J Clin Epidemiol 1990;43(6):551–8. 10.1016/0895-4356(90)90159-M 2189948

[21] Lin LI-K . A concordance correlation coefficient to evaluate reproducibility. Biometrics 1989;45(1):255–68. 10.2307/2532051 2720055

[22] Lin LI-K . A note on the concordance correlation coefficient. Biometrics 2000;56(1):324–5.

[23] McBride GB . A proposal for strength-of-agreement criteria for Lin’s Concordance Correlation Coefficient; 2005. NIWA Client Report: HAM2005-062. Hamilton (NZ): National Institute of Water and Atmospheric Research Ltd. https://www.medcalc.org/download/pdf/McBride2005.pdf. Accessed November 20, 2017.

[24] Hill EM , Turner EL , Martin RM , Donovan JL . “Let’s get the best quality research we can”: public awareness and acceptance of consent to use existing data in health research: a systematic review and qualitative study. BMC Med Res Methodol 2013;13(1):72. 10.1186/1471-2288-13-72 23734773 PMC3682867

[25] Rodriguez HP , Glenn BA , Olmos TT , Krist AH , Shimada SL , Kessler R , Real-world implementation and outcomes of health behavior and mental health assessment. J Am Board Fam Med 2014;27(3):356–66. 10.3122/jabfm.2014.03.130264 24808114 PMC4237013

[26] Tisnado DM , Adams JL , Liu H , Damberg CL , Chen WP , Hu FA , What is the concordance between the medical record and patient self-report as data sources for ambulatory care? Med Care 2006;44(2):132–40. 10.1097/01.mlr.0000196952.15921.bf 16434912

[27] Singleterry J , Jump Z , DiGiulio A , Babb S , Sneegas K , MacNeil A , State Medicaid coverage for tobacco cessation treatments and barriers to coverage — United States, 2014–2015. MMWR Morb Mortal Wkly Rep 2015;64(42):1194–9. 10.15585/mmwr.mm6442a3 26513425

[28] Sallis R , Franklin B , Joy L , Ross R , Sabgir D , Stone J . Strategies for promoting physical activity in clinical practice. Prog Cardiovasc Dis 2015;57(4):375–86. 10.1016/j.pcad.2014.10.003 25459975

[29] Krist AH , Glenn BA , Glasgow RE , Balasubramanian BA , Chambers DA , Fernandez ME , ; MOHR Study Group. Designing a valid randomized pragmatic primary care implementation trial: the my own health report (MOHR) project. Implement Sci 2013;8(1):73. 10.1186/1748-5908-8-73 23799943 PMC3694031

[30] Estabrooks PA , Boyle M , Emmons KM , Glasgow RE , Hesse BW , Kaplan RM , Harmonized patient-reported data elements in the electronic health record: supporting meaningful use by primary care action on health behaviors and key psychosocial factors. J Am Med Inform Assoc 2012;19(4):575–82. 10.1136/amiajnl-2011-000576 22511015 PMC3384114

